# The Forced Magnetostrictions and Magnetic Properties of Ni_2_MnX (X = In, Sn) Ferromagnetic Heusler Alloys

**DOI:** 10.3390/ma13092017

**Published:** 2020-04-25

**Authors:** Takuo Sakon, Junya Yamazaki, Takumi Komori, Takeshi Kanomata, Yasuo Narumi, Masayuki Hagiwara, Hiroyuki Nojiri, Yoshiya Adachi

**Affiliations:** 1 Department of Mechanical and Systems Engineering, Faculty of Science and Technology, Ryukoku University, Otsu 520-2194, Shiga, Japan; t170350@mail.ryukoku.ac.jp (J.Y.); t130251@mail.ryukoku.ac.jp (T.K.); 2 Research Institute for Engineering and Technology, Tohoku Gakuin University, Tagajo 985-8537, Miyagi, Japan; c1924007@mail.tohoku-gakuin.ac.jp; 3 Center for Advanced High Magnetic Field Science, Graduate School of Science, Osaka University, 1-1 Machikaneyama, Toyonaka 560-0043, Osaka, Japan; narumi@ahmf.sci.osaka-u.ac.jp (Y.N.); hagiwara@ahmf.sci.osaka-u.ac.jp (M.H.); 4 Institute for Materials Research, Tohoku University, Sendai 980-8577, Miyagi, Japan; nojiri@imr.tohoku.ac.jp; 5 Graduate School of Science and Engineering, Yamagata University, Yonezawa 992-8510, Yamagata, Japan; adachy@yz.yamagata-u.ac.jp

**Keywords:** ferromagnetic Heusler alloy, magnetostriction, magnetization, itinerant ferromagnetism, spin polarization

## Abstract

Experimental studies into the forced magnetostriction, magnetization, and temperature dependence of permeability in Ni_2_MnIn and Ni_2_MnSn ferromagnetic Heusler alloys were performed according to the spin fluctuation theory of itinerant ferromagnetism proposed by Takahashi. We investigated the magnetic field (*H*) dependence of magnetization (*M*) at the Curie temperature *T*_C_, and at *T* = 4.2 K, which concerns the ground state of the ferromagnetic state. The *M*-*H* result at *T*_C_ was analyzed by means of the *H* versus *M*^5^ dependence. At 4.2 K, it was investigated by means of an Arrott plot (*H*/*M* vs. *M*^2^) according to Takahashi’s theory. As for Ni_2_MnIn and Ni_2_MnSn, the spin fluctuation parameters in *k*-space (momentum space, *T*_A_) and that in energy space (frequency space, *T*_0_) obtained at *T*_C_ and 4.2 K were almost the same. The average values obtained at *T*_C_ and 4.2 K were *T*_A_ = 342 K, *T*_0_ = 276 K for Ni_2_MnIn and *T*_A_ = 447 K, *T*_0_ = 279 K for Ni_2_MnSn, respectively. The forced magnetostriction at *T*_C_ was also investigated. The forced linear magnetostriction (Δ*L*/*L*) and the forced volume magnetostriction (Δ*V*/*V*) were proportional to *M*^4^, which followed Takahashi’s theory. We compared the forced volume magnetostriction Δ*V*/*V* and mechanical parameter, bulk modulus *K*. Δ*V*/*V* is inversely proportional to *K*. We also discuss the spin polarization of Ni_2_MnIn and other magnetic Heusler alloys. The *p*_C_/*p*_S_ of Ni_2_MnIn was 0.860. This is comparable with that of Co_2_MnGa, which is a famous half-metallic alloy.

## 1. Introduction

Spin fluctuation theories have been proposed to explain the physical properties and the principles of itinerant electron systems [1,2,3,4,5,6,7]. Recently, the spin fluctuation theory of itinerant magnetism, known as Takahashi’s theory, was proposed by Takahashi [1,2,3,4]. The self-consistent renormalization (SCR) theory was first proposed by Moriya and Kawabata, taking into account the non-linear mode–mode coupling between spin fluctuation modes [5,6,7]. Concerned about the magnetic field dependence of magnetization (*M*–*H*), the effect of non-linear mode–mode couplings is associated with the second lowest expansion of free energy in regard to magnetization *M*. In this theory, the spin fluctuations of the higher order coefficient are neglected. Takahashi’s theory is the SCR theory according to zero-point spin fluctuations, considering the transverse and longitudinal components of the fluctuations. In this theory, the spin fluctuations of the higher order coefficient are considered, and the relationship between the magnetic fields *H* and magnetization *M* at *T*_C_ is obtained theoretically by Equation (1):

(1) $${\left(\frac{M}{Ms}\right)}^{4}=1.20\times 10^{6}\times \left(\frac{T_{C}^{2}}{w_{A}T_{A}^{3}p_{S}^{4}}\right)\times \left(\frac{H}{M}\right),$$

where *M*_S_ is spontaneous magnetization in the ground state, *p*_s_ is the magnetic moment in the ground state (*T* = 0 K), *T*_A_ is the spin fluctuation parameter in *k*-space (momentum space) in units of Kelvin, *w*_A_ is the molecular weight in units of g, and *H* is the magnetic field in units of kOe. Takahashi transcribed the spin fluctuation parameter in *k*-space at temperature *T*_A_ (K) [2]. The dynamical spin susceptibility, as shown in Equation (3.1) in reference [2], is demonstrated by the double-Lorentzian function of the *k*-space (parameter: *q*) and the energy space (frequency *ω*-space). The Lorentzian function of the *k*-space is proportional to *χ*(*q* = 0, *ω* = 0). The half-width of this function, Δ*q*, which indicates a spin fluctuation in *k*-space, is proportional to the inverse of *χ*(*q* = 0, *ω* = 0). The unit of 1/*χ*(*q* = 0, *ω* = 0) is a dimension of the energy. Finally, Δ*q* is shown in a dimension of the energy. Therefore, Δ*q* is proportional to *k*_B_*T*_A_, where *k*_B_ is the Boltzmann function and *T*_A_ is the spin fluctuation parameter, as mentioned above. *T*_A_ is expressed in the form of $T_{A}=\bar{A}q_{B}^{2}$, where $q_{B}^{2}$ indicates the effective zone boundary wave vector, and $\bar{A}$ indicates the non-dimensional parameter, as shown in Equation (3.6) in reference [2]. Another parameter, *T*_0_, is a spectral distribution *Γ*_qB_ in the frequency space, which was defined by *Γ*_qB_ = 2π*k*_B_*T*_0_. In this way, the spin fluctuation parameters in *k*-space (momentum space), *T*_A_, and that in energy space (frequency space), *T*_0_, were defined. From the spontaneous magnetic moment *M*_S_ and magnetization at *T*_C_, we obtained *T*_A_. Investigations into the itinerant magnetism of 3*d* and 5*f* electron systems were carried out by means of Equation (1) [1,8,9,10,11,12,13]. Moreover, this theory has been applied to the ferromagnetic Heusler alloys [11,14,15,16,17]. The spin fluctuation parameter in energy space *T*_0_ is derived from Equation (3.16) in reference [1]:

(2) $$p_{S}^{2}=\frac{20T_{0}}{T_{A}}\times C_{4/3}\times {\left(\frac{T_{C}}{T_{0}}\right)}^{4/3},\;C_{4/3}=1.006089\ldots .$$

From Equations (1) and (2), *T*_A_ and *T*_0_ are obtained.

The other method to derive the parameters *T*_A_ and *T*_0_ is determination from magnetic field dependence of the magnetization in the ground state (*T* << *T*_C_) [1,13,15].

The magnetization in the ground state is expressed by the following equation:

(3) $$H=\frac{F_{1}}{N_{0}^{3}{\left(g\mu_{B}\right)}^{4}}\times \left(-M_{0}^{2}+M^{2}\right)M,$$

where g indicates the Landé *g*-factor, *N*_0_ indicates Avogadro’s number, and *F*_1_ indicates the mode–mode coupling term of the spin fluctuations written as

(4) $$F_{1}=\frac{2T_{A}^{2}}{15cT_{0}}.$$

In Equation (4), *c* is equal to 1/2 and *M*_0_ is the spontaneous magnetization. Further, *F*_1_ is derived from the slope of the Arrott plot (*H*/*M* versus *M*^2^ plot) at low temperatures by Equation (5):

(5) $$F_{1}=\frac{N_{0}^{3}{\left(2\mu_{B}\right)}^{4}}{k_{B}\zeta },$$

where *k_B_* indicates the Boltzmann factor, and ζ indicates the slope of the Arrott plot (*M*^2^ versus *H*/*M*). Then, *T*_0_ and *T_A_* are provided by the following equations, respectively:

(6) $${\left(\frac{T_{C}}{T_{0}}\right)}^{5/6}=\frac{p_{S}^{2}}{5g^{2}C_{4/3}}\times {\left(\frac{15cF_{1}}{2T_{C}}\right)}^{1/2},$$

(7) $${\left(\frac{T_{C}}{T_{A}}\right)}^{5/6}=\frac{p_{S}^{2}}{5g^{2}C_{4/3}}\times {\left(\frac{2T_{C}}{15cF_{1}}\right)}^{1/2}.$$

These equations use units of kOe and emu/g for the magnetic fields *H* and magnetization *M*, respectively (p. 66 in reference [1]). The value of the magnetic fields *H* in 10 kOe is equal to the value in T (Tesla), and the value of magnetization *M* in emu/g is equivalent to the value in Am^2^/kg.

As for the itinerant ferromagnets, the relation between the effective magnetic moment *p*_eff_ and the spontaneous magnetic moment *p*_S_ can be expressed by a generalized Rhodes–Wohlfarth equation (Equation (3.47) in reference [1]):

(8) $$\frac{p_{eff}}{p_{S}}=1.4\times {\left(\frac{T_{O}}{T_{C}}\right)}^{2/3}.$$

Equation (8) can be rewritten as

(9) $$k_{m}=\left(\frac{p_{eff}}{p_{S}}\right)\times {\left(\frac{T_{C}}{T_{0}}\right)}^{\frac{2}{3}}.$$

Therefore, if *k*_m_ = 1.4, Equation (9) is equal to Equation (8).

The other characteristic property of Takahashi’s theory is that the forced volume magnetostriction Δ*V*/*V* and the magnetization *M* at *T*_C_ can be described as in reference [1]:

(10) $$\left(\Delta V/V\right)\propto M^{4},$$

where Δ*V*/*V* can be derived by the following equation:

(11) $$\left(\Delta V/V\right)=(\Delta L/L){/}_{/}+2\times {\left(\Delta L/L\right)}_{⊥},$$

where (Δ*L*/*L*)_//_ and ${\left(\Delta L/L\right)}_{⊥}$ are the forced linear magnetostriction parallel and perpendicular to an external magnetic field, respectively [18,19].

In this study, we selected Ni_2_MnIn and Ni_2_MnSn alloys. These alloys are ferromagnetic Heusler alloys and do not cause martensitic transformation [20], in contrast to Ni_2_MnGa with a martensitic transformation temperature *T*_M_ of 195 K [21]. These alloys have *L2*_1_-type cubic crystal structure. We considered the magnetostriction and magneto-volume effects of these alloys. We measured the forced longitudinal magnetostriction (Δ*L*/*L*)_//_ and ${\left(\Delta L/L\right)}_{⊥}$, derived the forced volume magnetostriction ΔV/V as shown by Equation (4), and evaluated the correlation between the magnetization and ΔV/V.

## 2. Materials and Methods

**Figure 1:**
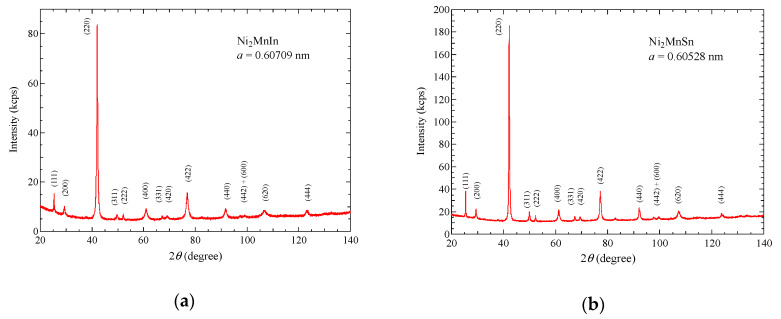
X-ray diffraction patterns of (**a**) Ni_2_MnIn and (**b**) Ni_2_MnSn. Parenthesis indicates the mirror indices.

Polycrystalline Ni_2_MnIn and Ni_2_MnSn alloys were synthesized from the constituent elements of NI_2_MnIn: Ni (4N), Mn (3N), In (4N); Ni_2_MnSn: Ni (4N), Mn (4N), Sn(5N). The sample of Ni_2_MnIn was prepared by induction melting under an Ar atmosphere. The sample of Ni_2_MnSn was prepared by arc-melting in an Ar atmosphere. The product of Ni_2_MnSn was heated in vacuum at 1123 K for 3 days and then quenched in water. The results of the X-ray diffraction pattern (XRD, Ultima IV, Rigaku Co., Ltd., Akishima, Tokyo, Japan) indicated that these samples were single phase, as shown in Figure 1. The XRD results indicated that the crystal structure is *L2*_1_ cubic, and lattice parameters *a* were 0.60709 nm and 0.60528 nm for Ni_2_MnIn and Ni_2_MnSn, respectively. A helium-free superconducting magnet at the High Field Laboratory for Superconducting Materials, Institute for Materials Research, Tohoku University, and at the Center for Advanced High Magnetic Field Science, Osaka University was used for the magnetostriction measurements up to 5 T. The magnetization measurement at 4.2 K, which corresponds to the investigation of the magnetic field dependence of the magnetization at the ground state (*T* << *T*_C_) was performed by means of 30 T pulsed field magnet at the Center for Advanced High Magnetic Field Science, Osaka University. A detailed explanation of the experimental procedure has been given in previous studies [14,15,16,17].

## 3. Results and Discussion

### 3.1. Magnetic Field Dependence of Magnetization

**Figure 2:**
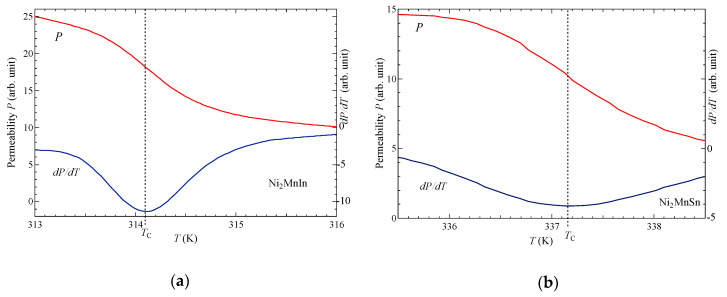
Permeability (*P*) and *dP*/*dT* (differential of the permeability in the temperature) of (**a**) Ni_2_MnIn and (**b**) Ni_2_MnSn around *T*_C_. The dotted lines define *T*_C_.

Figure 2 shows the temperature dependence of the permeability *P* for (a) Ni_2_MnIn and (b) Ni_2_MnSn in a zero external magnetic field. The values of *dP*/*dT* shown in Figure 2 are the values of the differential of the permeability in the temperature. For Ni_2_MnIn and Ni_2_MnSn, the values of *T*_C_ were obtained from the peak of *dP*/*dT*, which were 314 K and 337 K, respectively, using the same approach [14].

**Figure 3:**
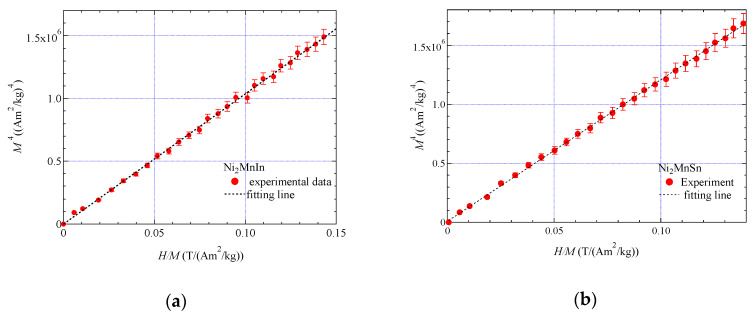
The magnetic field dependences of the magnetization, *M*^4^ vs. *H*/*M* at *T*_C_: (**a**) Ni_2_MnIn; (**b**) Ni_2_MnSn. Dotted straight lines are linearly fitting lines.

Figure 3 for (a) Ni_2_MnIn and (b) Ni_2_MnSn shows the plots of *M*^4^ versus *H*/*M* at *T*_C_. A good linearity can be seen at the origin at *T*_C_. The magnetic field dependence of the magnetization indicates that *H* ∝ *M*^5^; therefore, the results agree with Takahashi’s theory [1]. In former experimental investigations of Ni_2_MnGa-type Heusler alloys, such as Ni_2+*x*_MnGa_1−*x*_ (0≤x≤0.04) and Ni_2_Mn_1−*x*_Cr*_x_*Ga (0≤x≤0.25), Takahashi’s theory has also been adapted successfully [11,14,15,16,17]. The spin fluctuation parameter in *k*-space, *T*_A_, and in energy space, *T*_0_, has been calculated from the magnetization process at *T*_C_ using Equations (3) and (4) by Takahashi’s theory [1].

**Figure 4:**
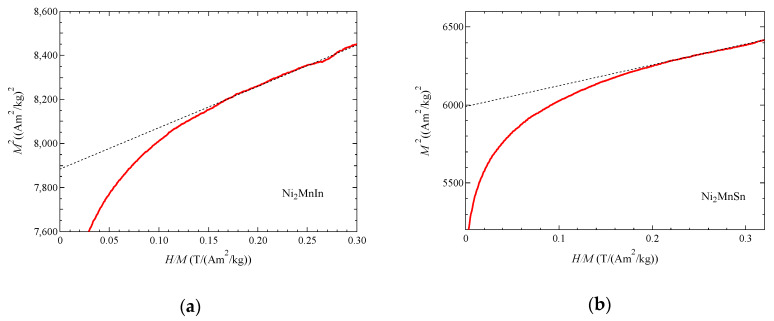
The magnetic field dependences of the magnetization, *M*^2^ vs. *H*/*M* at 4.2 K: (**a**) Ni_2_MnIn; (**b**) Ni_2_MnSn. Dotted straight lines are linearly fitting lines.

Furthermore, we investigated the magnetization measurement at 4.2 K, which corresponds to the magnetization process that was performed at the ground state (*T* << *T*_C_, *T*/*T*_C_ ≈1%). Figure 4 plots the magnetic field dependences of the magnetization, *M*^2^ versus *H*/*M*, which corresponds to the Arrott plot at 4.2 K for (a) Ni_2_MnIn and (b) Ni_2_MnSn [22]. These plots indicated that *M*^2^ was proportional to *H*/*M* in high magnetic fields and could be appreciable to Equation (3) of Takahashi’s theory [1]. Then, *T*_A_ and *T*_0_ were obtained by means of Equations (3)–(7).

The obtained parameters, *T*_A_ and *T*_0_, are listed in Table 1. These results indicate that Takahashi’s theory is applicable to Ni_2_MnIn and Ni_2_MnSn alloys. The experimental results followed the relation of $\left(\Delta V/V\right)\propto M^{4}$, which is correct in Equation (10), proposed by Takahashi’s theory [1].

### 3.2. Correlation between Magnetization and Forced Magnetostriction

**Figure 5:**
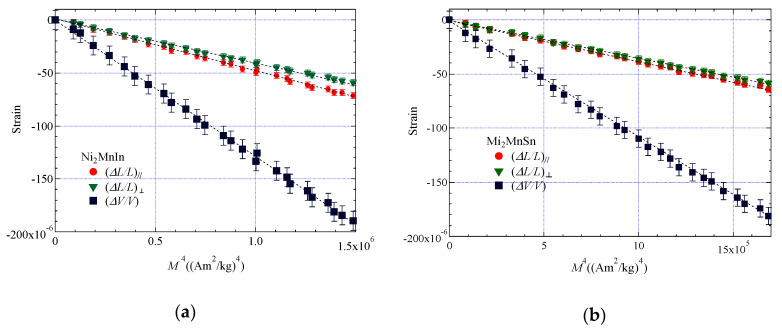
Forced magnetostriction vs. *M*^4^ at *T*_C_: (**a**) Ni_2_MnIn; (**b**) Ni_2_MnSn at *T*c. Dotted straight lines are linearly fitting lines.

In this subsection, we describe the investigations of forced magnetostrictions for Ni_2_MnIn and Ni_2_MnSn, and the correlation between forced volume magnetostriction and magnetization is discussed. In order to consider the relevance between magnetization and forced magnetostriction, we examined the magnetostriction in the magnetic fields and at *T*_C_. Figure 5 shows the external magnetic field dependence of the forced magnetostriction for (a) Ni_2_MnIn and (b) Ni_2_MnSn. The forced volume magnetostriction Δ*V*/*V* was derived using Equation (11). For both alloys, the obtained Δ*V*/*V* was proportional to the fourth power of the *M*, $\left(\Delta V/V\right)\propto M^{4}$, and crossed the origin, (*M*^4^, Δ*V*/*V*) = 0, as indicated by the dotted linearly fitting line. This result is consistent with other Ni_2_MnGa-type Heusler alloys [14,15,17]. Faske et al. conducted an experimental investigation into the magnetization *M* and magnetostriction Δ*V*/*V* of LaFe_11.6_Si_1.4_ [12]. They found the relationship between Δ*V*/*V* and *M* as $\left(\Delta L/L\right)\propto M^{4}$, and crossed the origin, and they suggested that the experimental results of Δ*V*/*V* and *M* were in accordance with Takahashi’s theory [1]. As for renowned weak ferromagnet MnSi [8], Takahashi suggested that the relationship between Δ*L*/*L* and *M* is $\left(\Delta L/L\right)\propto M^{4}$ [1]. Not only weak ferromagnet but also *L2*_1_-type cubic Heusler alloys, and LaFe_11.6_Si_1.4_ (NaZn13-type structure), which has a more complex structure, are in accordance with Takahashi’s theory.

In a previous study, we measured the magnetostrictions of Ni_2_MnGa-type and Heusler alloys at *T*_C_ and proved that Δ*V*/*V* is proportional to the valence electron per atom, *e*/*a* [17]. As for Ni_2_MnGa, Ni_2_MnIn, and Ni_2_MnSn, the *e*/*a* were all the same value as 7.500. Therefore, we compared the forced volume magnetostriction Δ*V*/*V* and its mechanical parameter, bulk modulus *K* [14,15]. The forced volume magnetostriction Δ*V*/*V* at 5 T and bulk modulus *K* are listed in Table 2. The *K* is inversely proportional to Young’s modulus. Therefore, as *K* becomes smaller, it softens more. The order of Δ*V*/*V* at 5 T is Ni_2_MnGa < Ni_2_MnSn < Ni_2_MnIn. The values of *M*^4^ for Ni_2_MnGa and Ni_2_MnIn are comparable. The *K* of Ni_2_MnIn is smaller than that of Ni_2_MnGa. Therefore, Ni_2_MnIn is softer than that of Ni_2_MnGa. It is conceivable that the strain grows larger for a softer alloy. Then, the Δ*V*/*V* of Ni_2_MnIn is larger than that of Ni_2_MnGa. The value of *M*^4^ for Ni_2_MnSn is larger than that of Ni_2_MnGa. Moreover, from the results of *K*, Ni_2_MnSn is softer than Ni_2_MnGa. Therefore, the Δ*V*/*V* of Ni_2_MnSn is larger than that of Ni_2_MnGa.

The units of *M*^4^ and *K* are defined by (Am^2^/kg)^4^ and Pa, respectively; Δ*V* and *V* are measured in m^3^; *K* is also defined in N/m^2^. The *K*Δ*V* is in the dimension of Pa·m^3^ = (N/m^2^)·m^3^ = Nm = J. Therefore, *K*·(Δ*V*/*V*) is in J/m^3^. Here, we defined the parameter *E*_K_ in J/m^3^. The Δ*V*/*V* = *E*_K_/*K*. This equation indicates that the forced volume magnetostriction Δ*V*/*V* is inversely proportional to bulk modulus *K*. The *K*·(Δ*V*/*V*) is also listed in Table 2. This is almost the same value. This result also indicates that Δ*V*/*V* is inversely proportional to *K*.

### 3.3. Spin Polarization of Ni_2_MnGa-Type Heusler Alloys

In this subsection, we consider the magnetism of Ni_2_MnGa-type Heusler alloys by comparing the spontaneous magnetic moment at the ground state, *p*_S_, and paramagnetic magnetic moment, *p*_C_.

The relation between *p*_eff_ and *p*_C_ is described as:

(12) $$p_{eff}=\sqrt{p_{C}\left(p_{C}+2\right)}.$$

The *p*_C_ is obtained from the Curie constant and it is non-dimensional, *C* = *N*_0_*μ*_eff_^2^/3*k*_B_ = *N*_0_*p*_eff_^2^*μ*_B_^2^/3*k*_B_ = *N*_0_*p*_C_(*p*_C_ + 2)*μ*_B_^2^/3*k*_B_. The *p*_c_/*p*_s_ is 1 for the local-moment ferromagnetism. For the weak itinerant electron ferromagnetism, the *p*_c_/*p*_s_ is larger than 1 [1]. On the contrary, many Heusler alloys have a *p*_c_/*p*_s_ value smaller than 1 [16]. As for the itinerant electron magnets, the minority-spin electrons band has a gap at the Fermi level *E*_F_ and indicates semi-metallic or insulating bands. On the contrary, the Fermi level intersects the majority-spin electrons band and represents metallic bands. The *p*_c_/*p*_s_ < 1 indicates that the spin polarization occurs, and these alloys can be classified as half-metallic alloys (HMFA). The *p*_S_ and *p*_C_ for Ni_2_MnGa-type Heusler alloys are listed in Table 3. Bocklage et al. performed point contact Andreev reflection (PCAR) spectroscopy on Ni_2_MnIn [26]. The obtained polarization value *P*_0_ was 35%. The *p*_C_/*p*_S_ of Ni_2_MnIn was 0.860. Both Co_2_VGa and Co_2_MnGa are known as typical HMAs. The *P*_0_ values were 75% and 48% for Co_2_VGa and Co_2_MnGa, respectively [27]. The *p*_C_/*p*_S_ values of Co_2_VGa and Co_2_MnGa were 0.70 and 0.80, respectively. The results for these three alloys indicate that the alloy with a larger spin polarization showed a smaller *p*_C_/*p*_S_ value. The spin polarization of Ni_2_MnSn was obtained by theoretical calculations [25]. The obtained *P*_0_ was about 10%, which indicates that the spin polarization of Ni_2_MnSn is smaller than that of Ni_2_MnIn. Then, the *p*_C_/*p*_S_ of Ni_2_MnSn was almost 1. Even at low temperature, Ni_2_MnIn and Ni_2_MnSn take an *L2*_1_-type cubic structure. On the contrary, Ni_2_MnGa causes martensitic transformation at *T*_M_ = 195 K, and below this temperature, 14 *M* structure was realized [28]. In the martensitic phase, the spin polarization was 19.72% [24]. Webster et al. analyzed the magnetic moment obtained by the saturation magnetization measurement, where *p*_S_ = 4.17 [29]. Then, the *p*_sat_/*p*_s_ was 0.92, which is smaller than 1 and deviated from 1 (local moment magnetism). The spin polarization of Ni_2_MnGa affected the deviation of the *p*_sat_/*p*_s_ value.

Takahashi’s theory can be applied even to the ferromagnetic Heusler alloy, which has a spin polarization, and further study is needed to clarify the origin of the magnetism and its physical properties.

.

## 4. Conclusions

In this article, we investigated the itinerant magnetism of Ni_2_MnIn and Ni_2_MnSn alloys. These alloys are ferromagnetic Heusler alloys and do not cause martensitic transformation [20], in contrast to Ni_2_MnGa with a martensitic transformation temperature *T*_M_ of 195 K [21]. These alloys have an *L2*_1_-type cubic crystal structure even at low temperature. We considered the magnetostriction and magneto-volume effects of these alloys. We measured the forced longitudinal magnetostriction (Δ*L*/*L*)_//_ and ${\left(\Delta L/L\right)}_{⊥}$, and we derived the forced volume magnetostriction ΔV/V. The correlation between the magnetization *M* and ΔV/V is $\left(\Delta L/L\right)\propto M^{4}$, and the linear fitting line crossed the origin for both alloys. These results were confirmed by Takahashi’s theory [1]. From the magnetization results at *T*_C_ and 4.2 K, the spin fluctuation parameters were *T*_A_ in *k*-space and *T*_0_ in energy space. The obtained *k*_m_ parameter of the generalized Rhodes–Wohlfarth equation was around 1.4. This result accorded with Takahashi’s theory. We considered the results of the examinations and theoretical calculations. We concluded that Takahashi’s theory can apply even to the ferromagnetic Heusler alloy, which has a spin polarization. We compared the forced volume magnetostriction Δ*V*/*V* and its mechanical parameter, bulk modulus *K*, and found that Δ*V*/*V* is inversely proportional to *K*.

## Figures and Tables

**Table 1:** Magnetic parameters of Ni_2_MnX (X = Ga, In, Sn). The spontaneous magnetic moment, *p*_S_; effective moment, *p*_eff_; Curie temperature, *T*_C_; spin fluctuation parameter in *k*-space, *T*_A_; spin fluctuation parameter in energy space, *T*_0_. The parameter *k*_m_ was obtained from Equation (9), which was almost the same as *k*_m_ = 1.4. “This work *T*_C_” indicates the values obtained from the magnetization process measurements at *T*_C_, and “This work 4.2 K” indicates the values obtained from the magnetization process measurements at 4.2 K.

Alloy	*p*_s_ (μ_B_/f. u.)	*p*_eff_ (μ_B_/f. u.)	*T*_C_ (K)	*T*_A_ (K)	*T*_0_ (K)	*k*_m_	Reference
Ni_2_MnGa	3.93	4.75	375	563	245	1.61	[15] *T* = *T*_C_
Ni_2_MnGa	3.93	4.75	375	556	254	1.57	[15] *T* = 5 K
Ni_2_MnIn	4.40 ^1^	4.69 ^2^	314	351	255	1.23	This work *T*_C_
Ni_2_MnIn	4.40 ^1^	4.69 ^2^	314	332	296	1.11	This work 4.2 K
Ni_2_MnSn	4.05 ^1^	5.00 ^2^	337	461	271	1.42	This work *T*_C_
Ni_2_MnSn	4.05 ^1^	5.00 ^2^	337	432	286	1.37	This work 4.2 K

^1^ [23], ^2^ [20].

**Table 2:** The forced volume magnetostriction Δ*V*/*V* at 5 T and the bulk modulus.

Alloy	Δ*V*/*V* at 5 T	*M*^4^ ((Am^2^/kg)^4^) at 5 T	Bulk Modulus *K* (GPa)	*K*·(Δ*V*/*V*) (J/m^3^)
Ni_2_MnGa	152 × 10^−6^ ^1^	1.52 × 10^6^ ^1^	166 ^2^	2.52 × 10^−2^
Ni_2_MnIn	190 × 10^−6^	1.49 × 10^6^	137 ^2^	2.60 × 10^−2^
Ni_2_MnSn	182 × 10^−6^	1.69 × 10^6^	143 ^3^	2.60 × 10^−2^

^1^ [14,15], ^2^ [24], ^3^ [25].

**Table 3:** Magnetic parameters of ferromagnetic Heusler alloys. *p*_C_ indicates the magnetic moment at the paramagnetic phase. The relationship between *p*_eff_ and *p*_C_ is defined by the equation of $p_{eff}=\sqrt{p_{C}\left(p_{C}+2\right)}$

Sample	*T*_C_ (K)	*p*_S_ (μ_B_/f.u.)	*p*_eff_ (μ_B_/f.u.)	*p*_C_ (μ_B_/f.u.)	p_C_/p_S_	Reference
Ni_2_MnGa	375	3.93	4.75	3.85	0.980	[16,20]
Ni_2_MnIn	314 *	4.4	4.69	3.78	0.860	* This work, [20]
Ni_2_MnSn	337 *	4.05	5.00	4.10	1.01	* This work, [20]

## References

[1] Takahashi Y. (2013). Spin Fluctuation Theory of Itinerant Electron Magnetism.

[2] Takahashi Y. (1986). On the origin of the Curie Weiss law of the magnetic susceptibility in itinerant electron ferromagnetism. J. Phys. Soc. Jpn..

[3] Takahashi Y. (2017). Theoretical Development in Itinerant Electron Ferromagnetism. J. Phys. Conf. Ser..

[4] Takahashi Y., Nakano H. (2006). Magnetovolume effect of itinerant electron ferromagnets. J. Phys. Cond. Matter..

[5] Moriya T. (1985). Spin Fluctuations in Itinerant Electron Magnetism.

[6] Moriya T., Kawabata A. (1973). Effect of Spin Fluctuations on Itinerant Electron Ferromagnetism. J. Phys. Soc. Jpn..

[7] Moriya T., Kawabata A. (1973). Effect of Spin Fluctuations on Itinerant Electron Ferromagnetism. II. J. Phys. Soc. Jpn..

[8] Matsunaga M., Ishikawa Y., Nakajima T. (1982). Magneto-volume effect in the weak itinerant ferromagnet MnSi. J. Phys. Soc. Jpn..

[9] Rizal C., Kolthammer J., Pokharel R.K., Choi B.C. (2013). Magnetic properties of nanostructured Fe-Co alloys. J. Appl. Phys..

[10] Nishihara H., Harada T., Kanomata T., Wada T. (2012). Magnetization process near the Curie temperature of an itinerant ferromagnet CoS_2_. J. Phys. Conf. Ser..

[11] Nishihara H., Komiyama K., Oguro I., Kanomata T., Chernenko V. (2007). Magnetization processes near the Curie temperatures of the itinerant ferromagnets, Ni_2_MnGa and pure nickel. J. Alloys Compd..

[12] Faske T., Radulov L.A., Hölzel M., Gutfleisch O., Donner W. (2020). Direct Observation of Paramagnetic Spin Fluctuations in LaFe_13−*x*_Si*_x_*. J. Phys. Condens. Matter..

[13] Tateiwa N., Pospíšil J., Haga Y., Sakai H., Matsuda T.D., Yamamoto E. (2017). Itinerant ferromagnetism in actinide 5*f*-electron systems: Phenomenological analysis with spin fluctuation theory. Phys. Rev. B.

[14] Sakon T., Hayashi Y., Fujimoto N., Kanomata T., Nojiri H., Adachi Y. (2018). Forced magnetostriction of ferromagnetic Heusler alloy Ni_2_MnGa at the Curie temperature. J. Appl. Phys..

[15] Sakon T., Hayashi Y., Li D.X., Honda F., Oomi G., Narumi Y., Hagiwara M., Kanomata T., Eto T. (2018). Forced Magnetostrictions and Magnetizations of Ni_2+*x*_MnGa_1−*x*_ at Its Curie Temperature. Materials.

[16] Sakon T., Hayashi Y., Fukuya A., Li D., Honda F., Umetsu R.Y., Xu X., Oomi G., Kanomata T., Eto T. (2019). Investigation of the Itinerant Electron Ferromagnetism of Ni_2+*x*_MnGa_1−*x*_ and Co_2_VGa Heusler Alloys. Materials.

[17] Sakon T., Yamasaki Y., Kodama H., Kanomata T., Nojiri H., Adachi Y. (2019). The Characteristic Properties of Magnetostriction and Magneto-Volume Effects of Ni_2_MnGa-Type Ferromagnetic Heusler Alloys. Materials.

[18] Kittel C. (2004). Introduction of Solid State Physics.

[19] Nizhankovskii V.I. (2006). Classical magnetostriction of nickel in high magnetic field. Eur. Phys. J. B.

[20] Kanomata T., Shirakawa K., Kaneko T. (1987). Effect of Hydrostatic Pressure on the Curie Temperature of the Heusler alloys Ni_2_MnZ (Z = AI, Ga, In, Sn AND Sb). J. Magn. Magn. Mater..

[21] Sakon T., Fujimoto N., Kanomata T., Adachi Y. (2017). Magnetostriction of Ni_2_Mn_1−*x*_Cr*_x_*Ga Heusler Alloys. Metals.

[22] Arrott A., Noakes J.E. (1967). Approximate equation of the state for Nickel near its Critical Temperature. Phys. Rev. Lett..

[23] Webster P.J. (1969). Heusler alloys. Contemp. Phys..

[24] Qawasmeh Y., Hamad B. (2012). Investigation of the structural, electronic, and magnetic properties of Ni based Heusler alloys from first principles. J. Appl. Phys..

[25] Jezierski A. (2019). Electronic structure, magnetic, optical and thermodynamic properties of Ni_2_Mn_1−*x*_Re*_x_*Sn and NiMn_1−*x*_Re*_x_*Sn Heusler alloys—Ab-initio study. J. Alloys Compds..

[26] Bocklage L., Scholtyssek J.M., Merkt U., Meier G. (2017). Spin polarization of Ni_2_MnIn and Ni_80_Fe_20_ determined by point-contact Andreev spectroscopy. J. Appl. Phys..

[27] Umetsu R.Y., Kobayashi K., Fijita A., Kainuma R., Ishida K., Fukamichi K., Sakuma A. (2008). Magnetic properties, phase stability, electric structure, and half-metallicity of *L2*_1_-type Co_2_ (V_1−*x*_Mn*_x_*)Ga Heusler alloys. Phys. Rev. B.

[28] Singh S., Bednarcik J., Barman S.R., Felsher S.R., Pandey D. (2015). Premartensite to martensite transition and its implications for the origin of modulation in Ni2MnGa ferromagnetic shape-memory alloy. Phys. Rev. B.

[29] Webster P.J., Ziebeck K.R.A., Town S.L., Peak M.S. (1984). Magnetic order and phase transition in Ni_2_MnGa. Philos. Mag. B.

